# Dipyrithione induces cell-cycle arrest and apoptosis in four cancer cell lines *in vitro* and inhibits tumor growth in a mouse model

**DOI:** 10.1186/2050-6511-14-54

**Published:** 2013-10-21

**Authors:** Yumei Fan, Caizhi Liu, Yongmao Huang, Jie Zhang, Linlin Cai, Shengnan Wang, Yongze Zhang, Xianglin Duan, Zhimin Yin

**Affiliations:** 1 The Key Lab of Animal Physiology, College of Life Science, Hebei Normal University, Hebei Province, Shijiazhuang 050024, China; 2 Laboratory of Medical Biotechnology, Hebei Chemical and Pharmaceutical College, Hebei Province, Shijiazhuang 050026, China; 3 Jiangsu Province Key Laboratory of Molecular and Medical Biotechnology, College of Life Science, Nanjing Normal University, Nanjing 210046, China

**Keywords:** PTS2, Anti-tumor activity, Chemotherapy

## Abstract

**Background:**

Dipyrithione (PTS2) is widely used as a bactericide and fungicide. Here, we investigated whether PTS2 has broad-spectrum antitumor activity by studying its cytotoxicity and proapoptotic effects in four cancer cell lines.

**Methods:**

We used MTT assays and trypan blue staining to test the viability of cancer cell lines. Hoechst 33258 and DAPI staining were used to observe cell apoptosis. Cell-cycle percentages were analyzed by flow cytometry. Apoptosis was assayed using caspase-3 and poly (ADP-ribose) polymerase (PARP) combined with Western blotting. Student’s *t*-test was used for statistical analysis.

**Results:**

PTS2 inhibited proliferation in four cancer cell lines in a dose-dependent manner. Treated cells showed shrinkage, irregular fragments, condensed and dispersed blue fluorescent particles compared with control cells. PTS2 induced cycle-arrest and death. Cleavage of caspase-9, caspase-3, and PARP were detected in PTS2-treated cells. Antitumor activity of PTS2 was more effective against widely used cancer drugs and its precursor.

**Conclusions:**

PTS2 appears to have novel cytotoxicity and potent broad-spectrum antitumor activity, which suggests its potential as the basis of an anticancer drug.

## Background

Apoptosis is a cellular progression characterized by a series of tightly regulated molecular processes leading to cell death [1]. There are two independent apoptosis pathways: the death receptor pathway and the mitochondria pathway [2], both of which converge on a family of cysteine aspartases called caspases, whose activity drives the biochemical events leading to cellular disassembly and death. Novel second mitochondria-derived activator of caspases (Smac) mimetic compounds sensitize human leukemia cell lines to conventional chemotherapies that induce death receptor-mediated apoptosis [3,4]. During apoptosis, cytochrome *c*, a component of the mitochondrial electron transfer chain, releases from mitochondria to the cytosol, and binds to Apaf-1, a cytosolic protein, forming the Apaf-1/cytochrome *c* complex, which then oligomerizes and forms apoptosomes by recruiting multiple procaspase-9 molecules, and then cleaving and activating downstream apoptosis effectors such as caspase-3, and PARP [5]. As malignancies grow, cancer cells evolve around mechanisms that limit cell proliferation, such as apoptosis and replicative senescence. Successful cancer therapies may trigger tumor-selective apoptosis [6].

**Figure 1:**
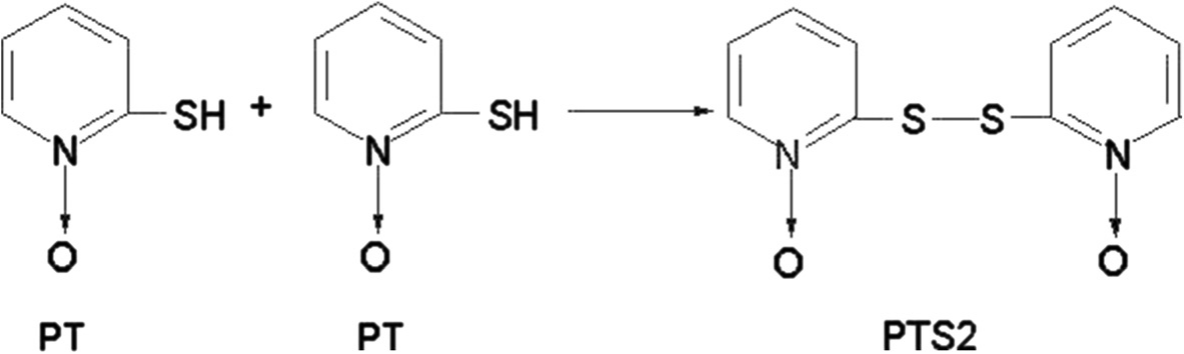
Chemical structure of PTS2.

Pyrithione (2-pyridinethiol-1-oxide, PT) has been used as a bactericide and fungicide for more than 50 years [7]. PT derivatives, such as zinc PT and sodium PT, are widely used as cosmetic preservatives and as anti-dandruff agents in shampoos. Zinc PT can reportedly induce apoptosis because of its role as a zinc-ionophore [8,9]. Compounds containing -SH group are quickly oxidized to generate disulfide. For PT, such self-oxidation would result in the formation of the dimer, 2,2’-dithiobispyridine-1,1’-dioxide (dipyrithione, PTS2; Figure 1), which also possesses anti-bacterial and anti-fungal activity. Our previous study demonstrated the cytotoxicity and effect of PTS2 in HeLa cells [4], and PTS2 inhibited inflammatory responses induced by lipopolysaccharides (LPS) in RAW264.7 cells, thus protecting mice against endotoxic shock by exerting anti-inflammatory effects through decreased formation of chemokine IP-10/CXCL10 and reduced acute oleic acid-induced lung injury [10-12].

Here we reported novel toxicity, including inhibited proliferation and induced apoptosis, of PTS2 in four cancer cell lines. Our results indicated that PTS2 has broad-spectrum antitumor activity, suggesting its potential as the basis of an anticancer drug.

## Methods

### Cell culture

MDA-MB-231 (human breast cancer cell line), KB (nasopharyngeal carcinoma cell line), U937 (human monoblast leukemia cell line), and K562 (human leukemia cell line) were purchased from the CBCAS (Cell Bank of the Chinese Academic of Sciences, Shanghai, China). Cells were maintained in RPMI1640 (GIBCO), supplemented with 10% (v/v) fetal bovine serum (HyClone), sodium bicarbonate, 100 μg/ml streptomycin and 100 U/ml penicillin (HyClone) at 37°C, in a humidified 5% CO_2_ atmosphere.

### Antibodies and reagents

PTS2 and PT were purchased from J&K Chemical LTD. Adriamycin (ADM) was purchased from Hisun (Zhejiang Hisun Pharceutical Co., LTD). Cisplatin (DDP) was purchased from QiLu (QiLu Pharmaceutical Co., LTD.). MTT, Hoechst33258, DAPI and propidium iodide (PI) were from Sigma (Sigma Chemical Co., St Louis, MO). Antibodies to caspase-3, PARP, CyclinD1, CyclinE1 and caspase-9 were purchased from Cell Signaling Technology (Beverly, MA). Antibodies to p21 were purchased from Santa Cruz Biotechnology (Santa Cruz, CA). All chemicals and drugs were prepared in PBS immediately before use.

### Western blotting

Western blotting was performed as described previously [13]. Cells were washed twice with ice-cold PBS (pH 7.4) and lysed in a lysis buffer containing 50 mM Tris–HCl (pH 8.0), 150 mM NaCl, 0.5 mM dithiothreitol, 1 mM EDTA, 1% NP-40, 10% (v/v) glycerol, 50 μg/ml phenylmethylsulfonyl fluoride, 2 μg/ml aprotinin, 1 μg/ml leupeptin, 1 μg/ml pepstatin and 1 mM Na_3_VO_4_. After incubation on ice for 20 min, lysates were centrifuged at 15,000× g for 10 min at 4°C and the supernatant was transferred to a clean microfuge tube. Equal amounts of the soluble protein were denatured in SDS, electrophoresed on SDS-polyacrylamide gel, and transferred to a PVDF membrane. Horseradish peroxidase (HRP)-conjugated goat anti-rabbit IgG antibodies were used against respective primary antibodies. Proteins were visualized using Lumi-Light Western Blotting Substrate (Roche Molecular Biochemicals). The total density of the protein bands was calculated using the Scion Image software program (Scion Corp., Frederick, MD).

### Cell proliferation assay

Cells were seeded into 96-well plates at 5 × 10^3^ cells per well 24 h before treatment. After treatment with different drugs, cell proliferation was determined using MTT (3-(4,5 dimethylthiazol-2-yl)-2,5-diphenyltetrazolium bromide) assay. Briefly, 15 μl (5 mg/ml) MTT solution was added to each well, and incubated at 37°C for 4 h, after which the MTT solution was removed and 200 μl of dimethylsulfoxide (DMSO) added to dissolve the crystals. Absorbance of each well was measured at 570 nm using an ELx 800 Universal Microplate Reader (Bio-Tek, Inc.) according to manufacturer’s instructions.

### Trypan blue assay

Cells were seeded in 6-well culture plates. After 24 h, culture medium containing 2.5 μg/ml of PTS2 was added to the wells. Cells were harvested at indicated times and washed with PBS, followed by centrifugation at 2500 g for 5 min. The cell pellet was then re-suspended in 1 ml of fresh culture medium; 10 μl of the cell suspension was stained with an equal volume of trypan blue (Sigma, Germany) and incubated for 2 min at 37°C. The total number of viable cells was estimated using a hemocytometer chamber.

### Hoechst33258 Staining

A staining solution of Hoechst33258 was prepared immediately before use. After drug treatment, cells were collected and fixed in acetic acid/methanol (1:3) solution for 5 min at 4°C, washed 3 times with PBS, and then incubated with Hoechst33258 (50 ng /ml) for 5 min and washed 3 times with PBS. Cells were then assessed for Hoechst fluorescence in a Nikon Optiphot fluorescence microscope (magnification: ×400).

### DAPI staining

Cells for 4′-6-diamidino-2-phenylindole (DAPI) staining underwent the same PTS2 treatment as those stained with Hoechst33258. Collected cells were fixed with acetic acid/methanol (1:3) solution for 10 min at room temperature and then incubated in DAPI (1 μg/ml) for 5 min. After being washed 3 times with PBS, cells were examined using a Nikon Optiphot fluorescence microscope (magnification: ×400).

### Cell cycle analysis

Cell cycle distribution was analyzed by flow cytometry. Control and treated cells were harvested, washed twice with PBS, and fixed in 70% ethanol overnight at -20°C. Fixed cells were washed twice with PBS, incubated with 1 ml of PBS containing 50 μg/ml propidium iodide, 100 μg/ml RNase A and 0.1% Triton X-100 for 30 min at 37°C. Stained cells were analyzed using a FAScan laser flow cytometer (Becton Dickinson) and ModFit LT cell cycle analysis software (Verity Software).

### Apoptosis assay

Apoptosis was determined using Annexin V-FITC/ PI double staining. After treatment, floating and adherent cells were collected, washed twice with PBS (pH 7.4), resuspended in 150 μl of Annexin-binding buffer and incubated with 0.4 μl of Annexin V-FITC. After 20 min incubation in the dark at room temperature, 150 μl of Annexin-binding buffer with 3 μl of PI (50 μg/ml) was added just before flow cytometry. Data were analyzed by flow cytometry using the FACSCalibur and Cell Quest software (Becton Dickinson).

### Animals and solid tumor models

All experiments followed the recommendations of the Chinese Experimental Animals Administration Legislation, as approved by the Science and Technology Department of Jiangsu Province. Male ICR mice (6 weeks old, 18–20 g) purchased from Shanghai Laboratory Animal Center, Chinese Academy Sciences, were kept in groups of five animals per cage in a temperature-controlled room at 20 ± 2°C. They were fed a standard pellet diet and water *ad libitum*. As described in [4], two groups of 40 animals each were transplanted subcutaneously with hepatoma 22 (H22) tumor cells (5 × 10^6^ cells/ml) in 0.2 ml PBS into their right groins. At 24 h after tumor inoculation, each set of 40 mice was randomly divided into 4 groups (10 mice per group) and injected intraperitoneally with PTS2 (0.25 or 2.5 mg/kg/day), DDP (25 mg/kg/day) or 0.2 ml 0.9% saline for a further 10 days. On day 11, all the animals were killed, and the tumors were dissected and weighed. Tumor growth inhibition was calculated using the formula:% inhibition = 100 × ([C - T]/C), where C is the average tumor weight of the control group and T is the average tumor weight of each treated group.

### Statistics

Statistical analysis used SPSS 12.0 (SPSS, Chicago, IL, USA). Results are expressed as means ± S.D. Differences between means were determined by one-way ANOVA, followed by Student–Newman–Keuls tests for multiple comparisons and Student’s *t* test for other data. *P* < 0.05 was considered statistically significant.

## Results

### PTS2 decreases cancer cell viability

**Figure 2:**
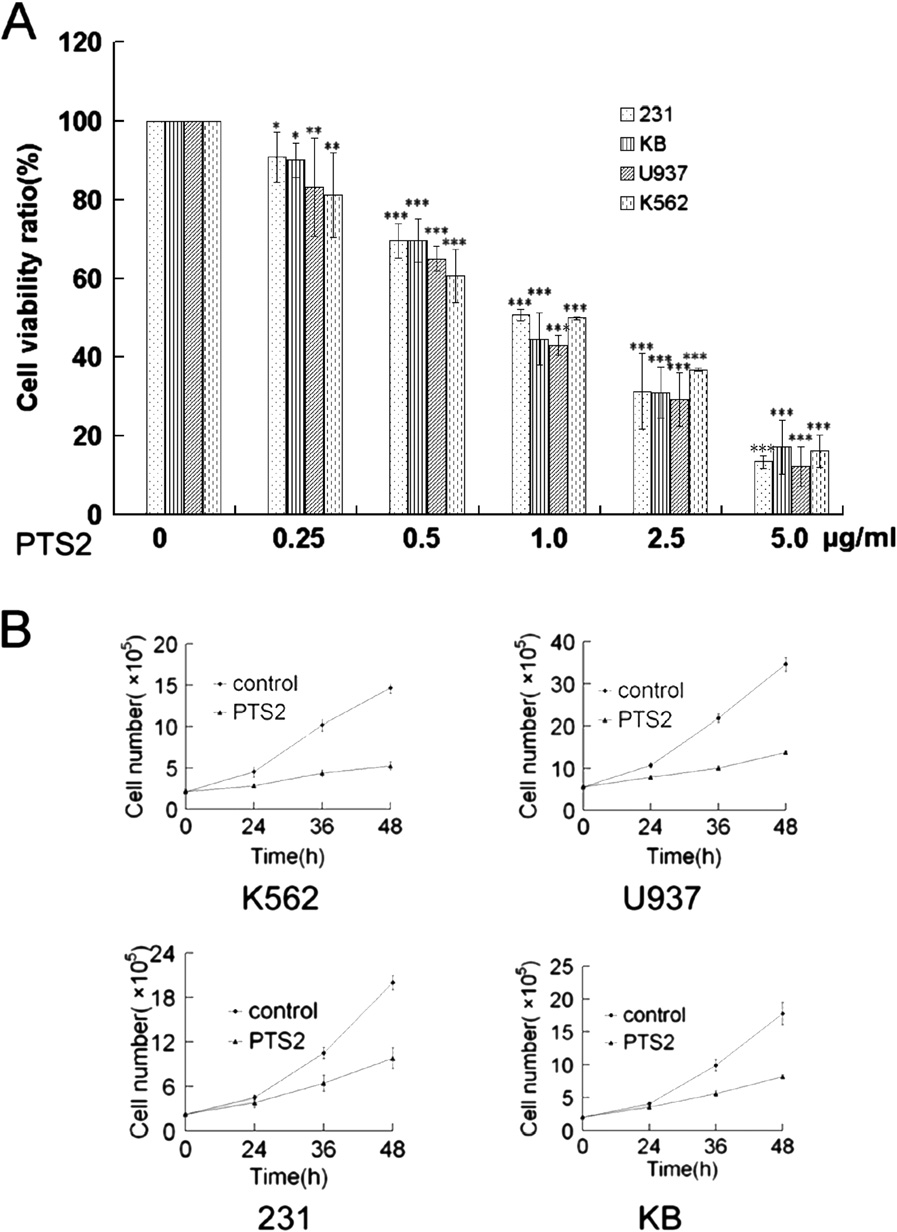
**Effects of PTS2 treatment on cancer cells viability. (A)** Four cancer cell lines were treated with indicated doses of PTS2 for 36 h. Cell viability was determined by MTT assay. Untreated cells were expressed as 100%. Data are means ± S.D. from 3 independent experiments. *P < 0.05; **P < 0.01; ***P < 0.001 compared with untreated controls. **(B)** Cancer cells were incubated with 2.5 μg/ml of PTS2, harvested at indicated times, and counted. Results show cell numbers increase over time. Data are means ± S.D. from 3 independent experiments.

To assess effects of PTS2 on cancer cell growth or proliferation, 4 cancer cell lines, including KB, 231, U937 and K562, were assayed using MTT. After 36 h treatment with various concentrations of PTS2, cell viability was determined for all 4 cell lines. PTS2 (0.25–5 μg/ml) was found to decrease cell viability in a dose-dependent manner (Figure 2A). The effect of PTS2 on cell proliferation was also examined. Results showed that PTS2 significantly decreased cell numbers in all 4 cancer cell lines compared with controls, as assessed using trypan blue (Figure 2B). These data indicate that PTS2 can decrease cell viability or proliferation within a suitable concentration range.

### PTS2 induces cell cycle arrest in cancer cells

**Figure 3:**
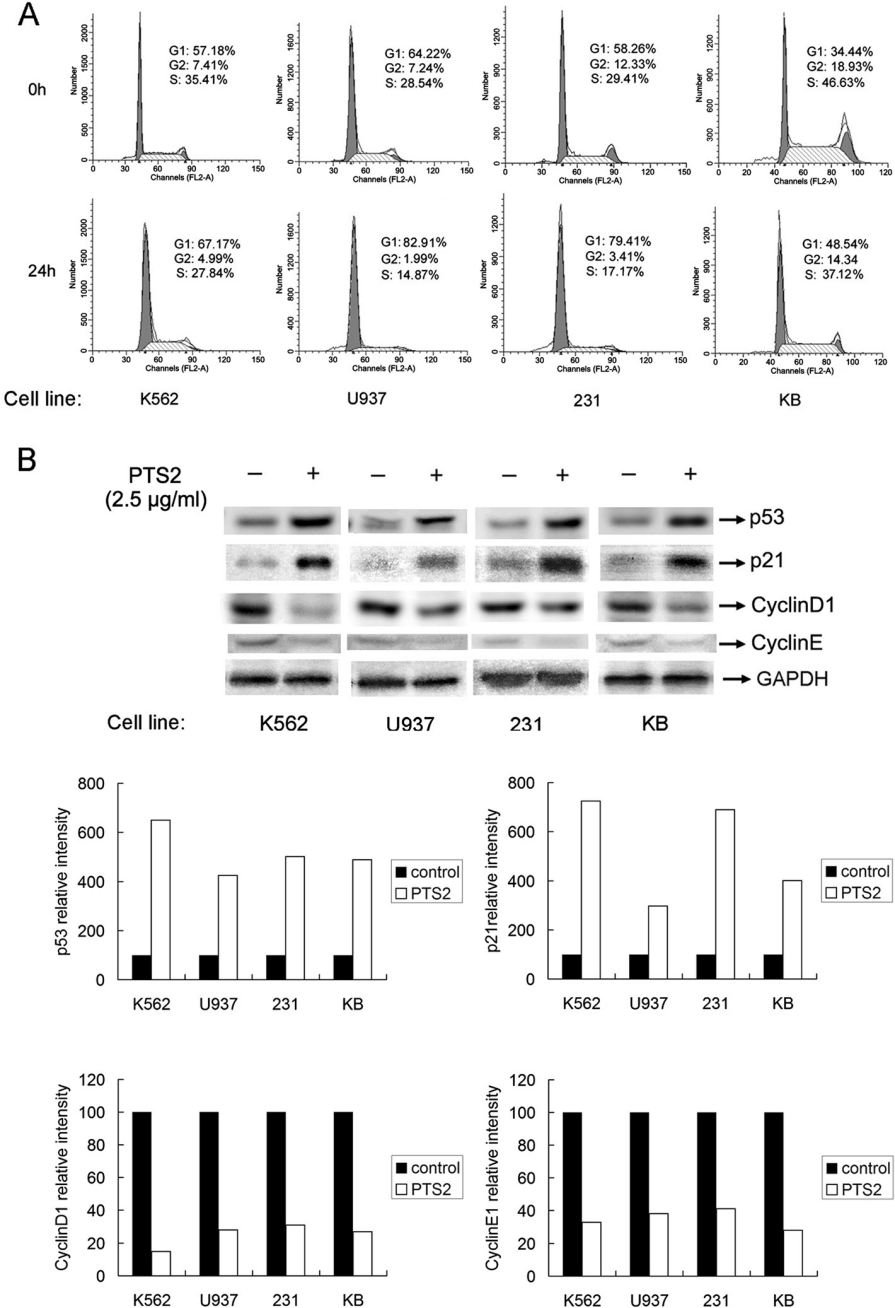
**PTS2 induces cell cycle arrest in cancer cells. (A)** Cancer cells were incubated with PTS2 (2.5 μg/ml) for 24 h, harvested and stained with propidium iodide (PI). Nuclei fluorescence was measured. Percentages of cells in each cell cycle phase are also shown. Results are representative of 3 independent experiments. **(B)** Cells were incubated with PTS2 (2.5 μg/ml) for 24 h, and then harvested. Western blot was performed using antibodies to p53, p21, CyclinD1, CyclinE1 and GAPDH. Results represent three independent experiments.

Cell proliferation is well correlated to regulation of cell cycle progression. A common mechanism for chemotherapeutic drugs is blocking passage through the G_1_ phase of the cell cycle [14]. We therefore investigated whether PTS2 can cause G_1_ cell cycle arrest in treated cancer cells (Figure 3). Cells exposed to PTS2 at 2.5 μg/ml for 24 h showed a marked increase in the percentage in G_1_ phase, and concomitant decreases in S phase and G_2_ phase populations, compared with vehicle-treated controls (Figure 3A).

As p21^WAF1/Cip1^ (p21) inhibits the cell cycle through its interaction with cyclin–CDK complexes [15], and is induced by p53 in response to DNA damage resulting in CDK inhibition and G_1_ growth arrest [16]. We further tested the effect of PTS2 on endogenous p53, p21, CyclinD1 and CyclinE1 expression in the four cancer cell lines. After cells were treated with PTS2 (2.5 μg/ml) for 24 h, PTS2 induced p21 accumulation in all cells as shown by Western blot (Figure 3B). CyclinD1 and CyclinE1 expressions were downregulated. These results were in accordance with the cell cycle experiment and previous reports [17,18], and suggest that PTS2-induced G_1_ arrest in cancer cells could be mediated via modulation of p53, p21, CyclinD1 and CyclinE1 levels.

### PTS2 induces cancer cell apoptosis

**Figure 4:**
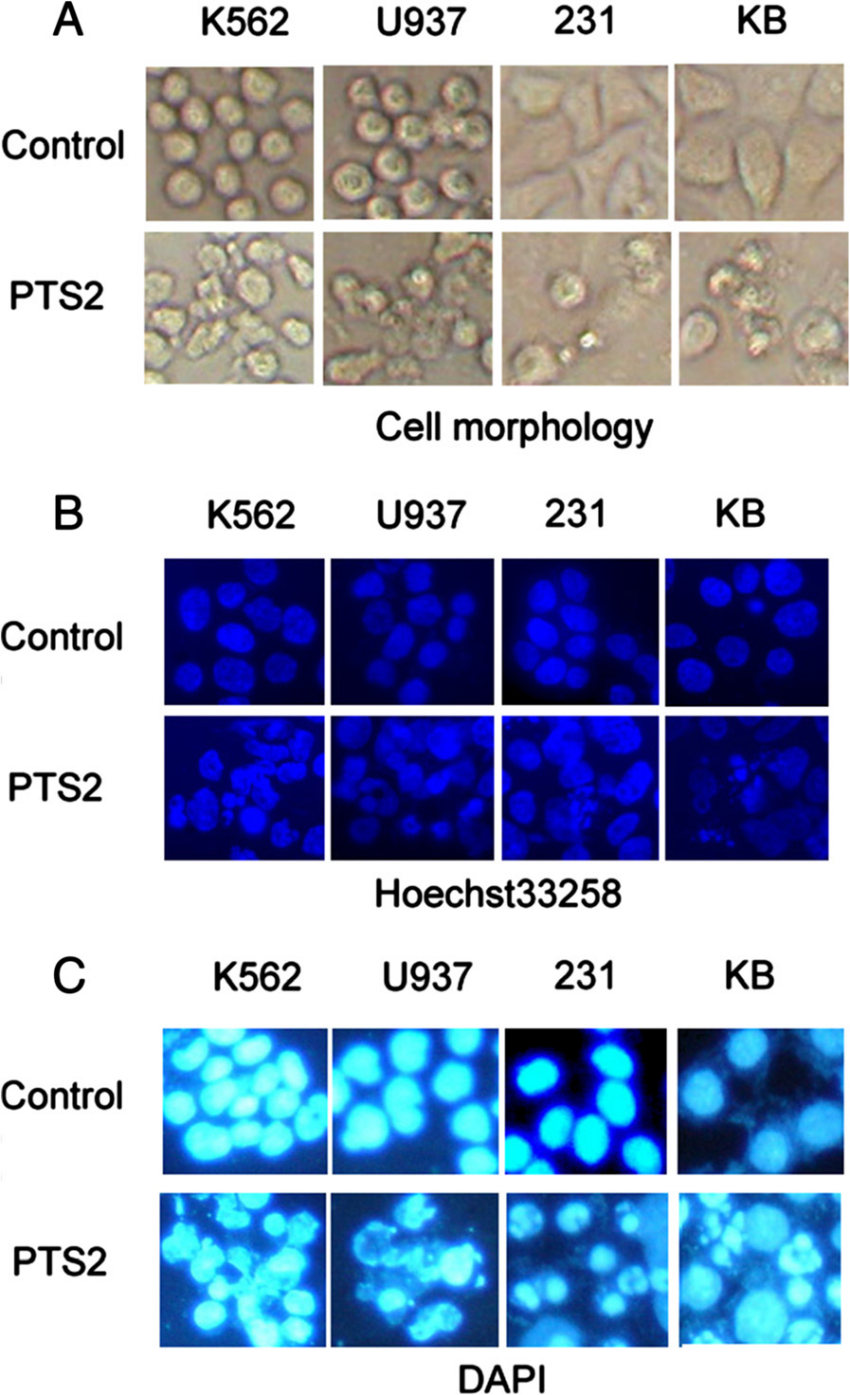
**Treatment with PTS2 induces apoptosis in cancer cells.** All 4 cancer cell lines were treated with PTS2 (2.5 μg/ml) for 36 h, along with untreated controls. Apoptotic nuclear fragmentation bodies, condensed and dispersed or fragmented particle were stained with blue fluorescence by Hoechst33258 (50 ng/ml) **(B)** and DAPI (1 μg/ml) **(C)**. Cell morphology **(A)** was observed in cells treated with PTS2 compared with control cells, using fluorescence microscopy (magnification: × 400).

Morphology alteration and chromatin condensation are two indicators of cell apoptosis. To verify whether the growth inhibitory effect of PTS2 was due to apoptosis, KB, 231, U937 and K562 cells were treated with PTS2 at 2.5 μg/ml for 36 h, and then observed under a light microscope or under a fluorescence microscope after Hoechst33258 and DAPI staining. We found the PTS2-treated cells were shrunken, with irregular fragments and apoptotic bodies, in contrast to control cells (Figure 4A). The typical apoptosis appearance, including condensed and dispersed or fragmented blue fluorescent particles was observed in PTS2-treated cells compared with control cells (Figure 4B, C), which suggests that PTS2 induces apoptosis in these types of cancer cells obviously.

### PTS2 induced cleavage of caspase-9, caspase-3 and PARP

**Figure 5:**
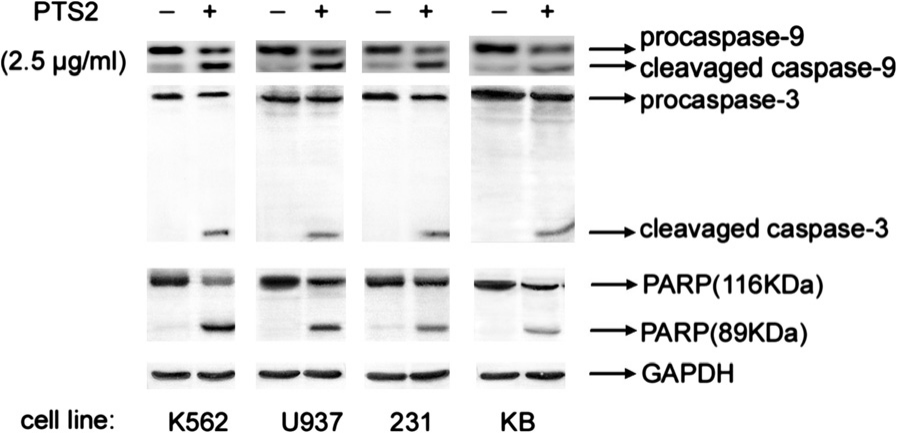
**Effects of PTS2 treatment on caspase-9, caspase-3 and PARP.** Cancer cells were treated with 2.5 μg/ml of PTS2 for 36 h, and then harvested. Western blot was used to determine proteolytic cleavage of caspase-9, caspase-3 and PARP. GAPDH was used as internal control. Results represent 3 independent experiments.

Caspase family members, including caspase-9 and caspase-3, as well as downstream substrates such as PARP, are crucial mediators of the apoptotic process. To see whether PTS2-induced cell death involved activation of caspases and PARP, we analyzed cleavage of caspase-9, caspase-3 and PARP in PTS2-treated cancer cells. Cells were incubated with 2.5 μg/ml of PTS2 for 36 h. As expected, Western blot results showed that caspase-9, caspase-3 and PARP were cleaved (Figure 5). Activation of caspases and PARP thereby confirmed apoptosis. This suggests that cleavage of caspase-9, caspase-3 and PARP are involved in PTS2-induced cancer cell apoptosis.

### PTS2 is more effective to induce cancer cell apoptosis against DDP, ADM or PT

**Figure 6:**
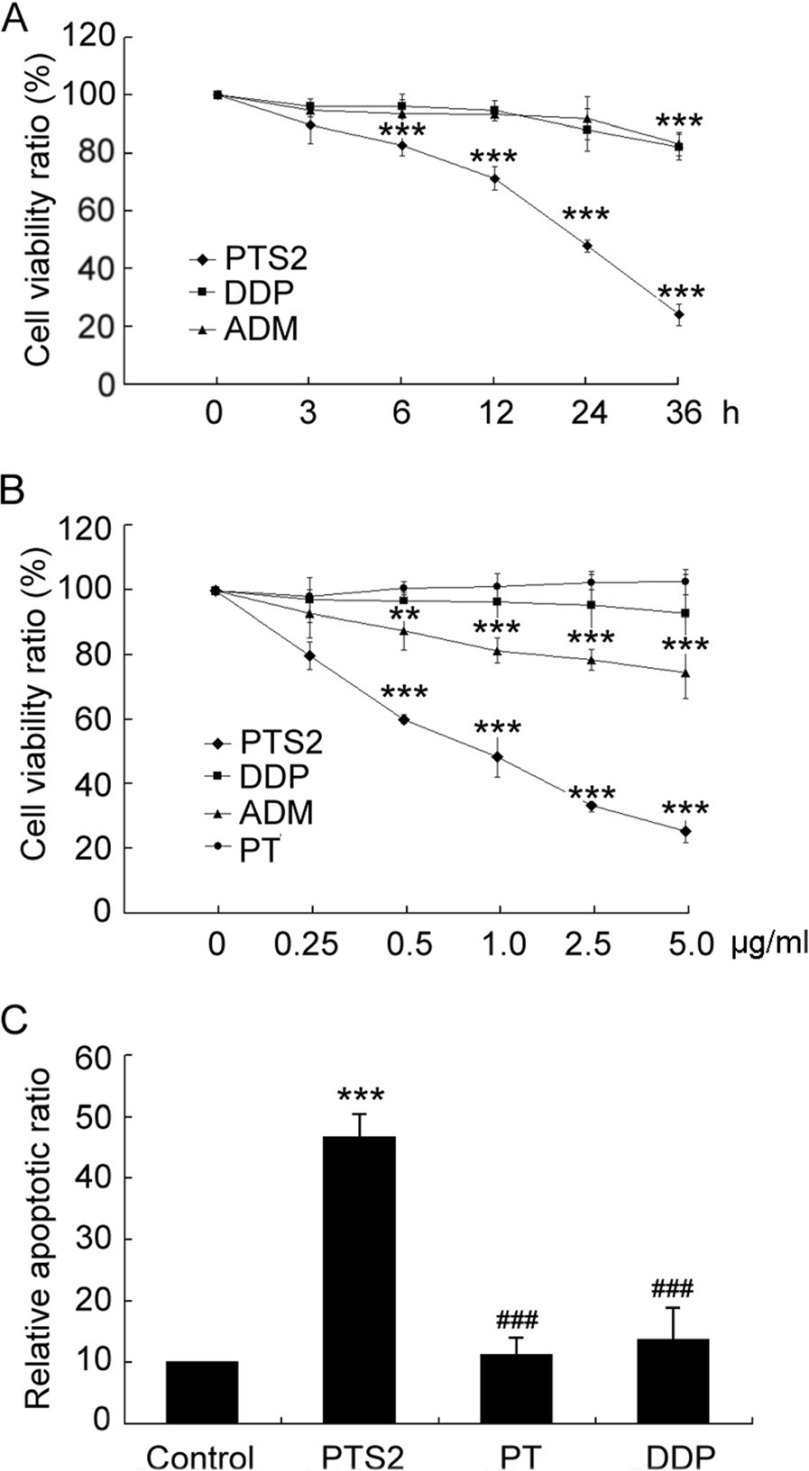
**Comparison of effects of PTS2, PT, DDP and ADM on KB cell viability. (A)** PTS2, PT, DDP and ADM (2.5 μg/ml) were added to KB cells, and assayed using MTT to determine cell viability after incubation for the indicated times. **(B)** In KB cells exposed to the indicated amounts of PTS2, PT, DDP and ADM for 36 h, cell viability was assayed using MTT. Data are means ± S.D. from 3 independent experiments. **P < 0.01; ***P < 0.001 compared with untreated controls. **(C)** KB cells were treated with 2.5 μg/ml of PTS2, PT, and DDP for 36 h, stained with Annexin V/PI, and examined with flow cytometry. Data are means ± S.D. ***P < 0.001, compared with viability of untreated cells. ^###^*P* < 0.001, compared with viability of PTS2-treated cells. Results are representative of 3 independent experiments.

Adriamycin (ADM) and cisplatin (DDP) are widely used cancer drugs. To further evaluate the efficacy of PTS2 in decreasing cancer cell viability, we compared its effects on KB cells to those of ADM and DDP. KB cells were exposed to 2.5 μg/ml of PTS2, ADM or DDP and assayed with MTT. At 3–36 h after treatment, PTS2 also showed faster results than did ADM or DDP (Figure 6A). In the dose range of 0.25–5.0 μg/ml, PTS2 was more effective in inhibiting cell viability than was ADM or DDP, although PT, the precursor of PTS2, exerted no obvious effect (Figure 6B). Flow cytometry analysis showed 2.5 μg/ml of PTS2 to be more effective in killing KB cell than PT and DDP (Figure 6C). These results strongly suggest that PTS2 can induce cancer cell death at least as efficiently as current anti-tumor drugs.

### PTS2 inhibits tumor growth

**Figure 7:**
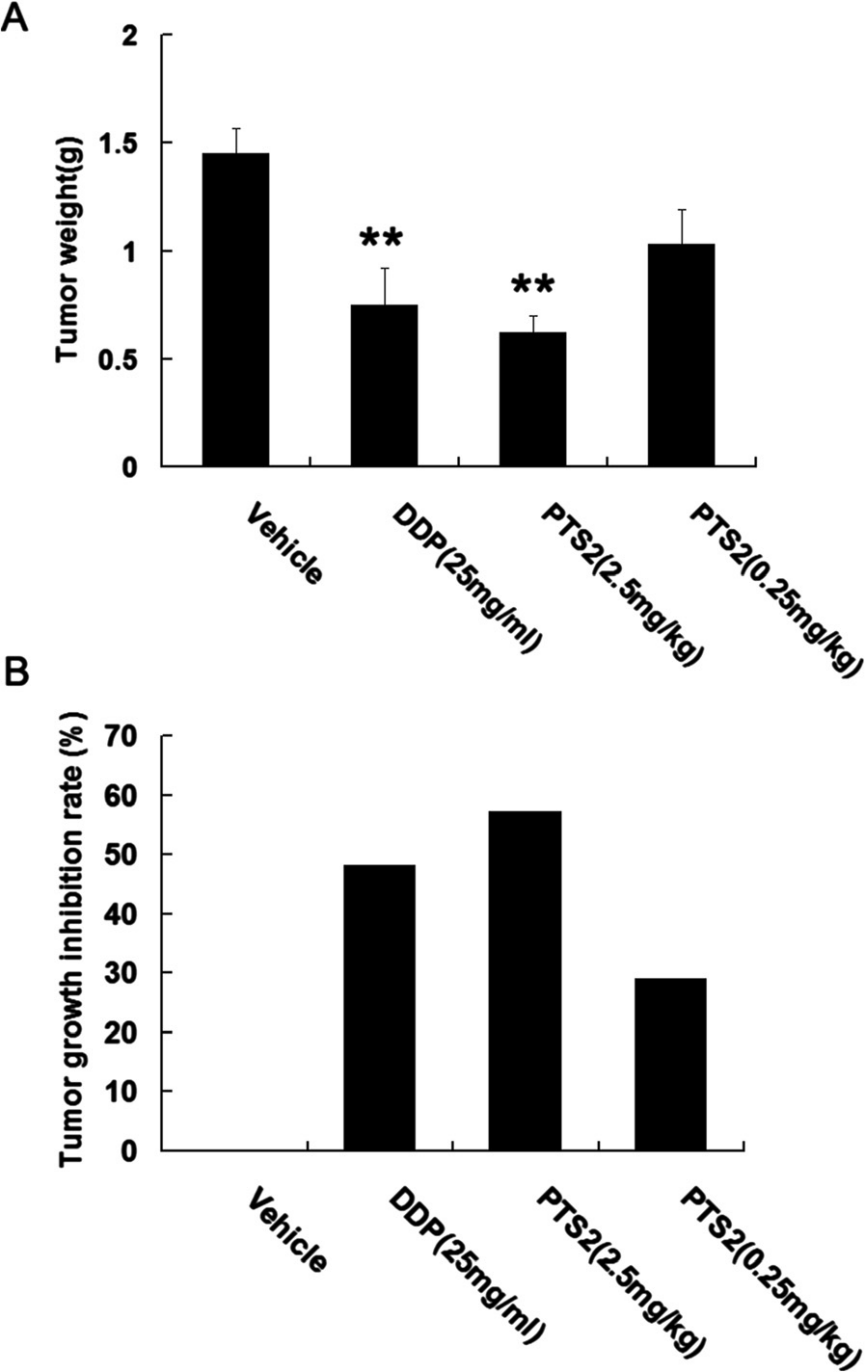
**Inhibitory effect of PTS2 on hepatoma 22 (H22) tumors.** H22 cells (5 × 10^6^ cells/ml) in 0.2 ml of PBS were transplanted subcutaneously into right groins of mice; 24 h after tumor implantation, the mice were injected intraperitoneally with PTS2 (0.25 or 2.5 mg/kg/day; n = 20), DDP (2.5 mg/kg/day; n = 10) or 0.2 ml 0.9% saline (n = 10) for another 10 days. On day 11, all the animals were killed and the tumors dissected out and weighed **(A)**. **P < 0.01 compared with controls. Tumor growth inhibition rate was calculated **(B)**.

To see effects of PTS2 on murine solid tumors, hepatoma 22 (H22) cells (5 × 10^6^ cells/ml) were transplanted subcutaneously into the right groins of mice; 24 h after tumor implantation, the mice were injected intraperitoneally with PTS2, DDP or saline and observed for 10 days. PTS2 at 2.5 mg/kg/day reduced the weight of H22 tumors; DDP had a similar effect at 25 mg/kg/day (Figure 7A). The tumor growth inhibition rate of PTS2 (2.5 mg/kg/day) was more effective than DDP (25 mg/kg/day) (Figure 7B). Moreover, the body weight increase of the PTS2-treated mice was greater than that of the DDP-treated mice (data not shown).

## Discussion

PTS2, a compound related to pyridinethione, has been used as a bactericide, pesticide and fungicide for a long time. Our previous report showed that PTS2 exerted cytotoxicity on HeLa cells and reduced the weight of S180 and H22 tumors [4]. The goal of the present study was to explore the cytotoxicity of PTS2 in other cancer cell lines and assess its potential broad-spectrum antitumor activity. We found PTS2 induced cell death in four cancer cell lines *in vitro*, and decreased viability in these cell lines in a dose-dependent manner (Figure 2). At a dose of 2.5 μg/ml, PTS2 induced apoptosis in various cancer cell lines, as detected by morphological and fluorescence analysis (Figure 4), thus raising the possibility that capsaicin might be a potential chemopreventive or therapeutic agent.

Many chemotherapeutic agents reportedly suppress cancer cell growth through disruption of cell cycle progression [19]. Upon cellular stress or DNA damage, these mechanisms induce cells to undergo either cell-cycle arrest, activation of repair systems, or apoptotic induction. In our present study, we showed that PTS2 evidently interferes with the cell cycle *in vitro* (Figure 3), arresting cells at G_1_ phase, and thus leading them to apoptosis. Presently, the molecular mechanisms of PTS2-induced cell cycle arrest in cancer cells require further investigation. Cells treated with PTS2 appear morphologically damaged and decreased in number (Figure 4). PTS2’s apparent induction of G_1_ arrest and subsequent apoptosis suggest that it could be the basis of an anticancer therapy. PTS2 can also induce p53, p21 accumulation and CyclinD1 and CyclinE1 downregulation in the four tested cancer cell lines (Figure 3B).

Caspase family members, including caspases 3 and 9, are crucial effectors of apoptosis, and are cleaved during apoptosis [19,20]. PARP is a substrate of caspase-3; its cleavage can indicate caspase activation in response to apoptotic stimulus [21,22], and generation of cleaved caspase-3 and PARP as markers for apoptosis [23]. In analyzing the mechanism of PTS2-induced apoptosis, we found PTS2 treatment induced cleavage of caspase-9, caspase-3 and PARP in cancer cells (Figure 5), which implies that activation of caspase-9, caspase-3 and PARP is involved in PTS2-induced cell death.

Such specific knowledge of PTS2’s anticancer effects is of great benefit in antitumor therapeutic strategy. PTS2 showed both dose and time advantages in inducing cancer cell death over adriamycin (an amino-glycosidic anthracycline antibiotic) and cisplatin (a platinum compound), which suggests PTS2’s potential as an anticancer drug (Figure 6). Although both PT and its derivative, PTS2, have antifungal activities [24], PT showed no obvious anticancer activity in this study.

## Conclusions

Here, we report novel toxicity of PTS2 on various cancers, and show PTS2 to inhibit proliferation of four cancer cell lines and induce apoptosis involving activation of caspase-9, caspase-3 and PARP. These results suggest that PTS2 has broad-spectrum antitumor activity and could be the basis of an anticancer drug.

## Competing interests

The authors declare that they have no competing interests.

## Authors’ contributions

YF, CL and SW carried out the experimental studies. YF and YZ drafted and completed the manuscript. YH performed the statistical analysis. JZ and LC cultured cells. ZY proofread the manuscript. XD and ZY refined the manuscript. FY and XD conceived of and designed the study. All authors read and approved the final manuscript.

## Pre-publication history

The pre-publication history for this paper can be accessed here:

http://www.biomedcentral.com/2050-6511/14/54/prepub
